# Laparoscopy and methylene blue staining angiography for precise synchronous resection of small intestinal vascular malformations: a case report

**DOI:** 10.3389/fmed.2025.1621874

**Published:** 2025-08-21

**Authors:** Zhi-Wei Zhang, Jun-Qiang Chen

**Affiliations:** Department of Gastrointestinal Surgery, Jinhua Central Hospital, Jinhua, China

**Keywords:** chronic intestinal bleeding, laparoscopy, methylene blue staining, angiography, small intestinal vascular malformations

## Abstract

**Background:**

Chronic intestinal bleeding caused by vascular malformations is uncommon. Locating these small intestinal vascular malformations with precision during surgery remains a challenge. With the rapid development of digital subtraction angiography (DSA), the detection of small intestinal vascular malformations has become easier. However, heterochronous resection of the diseased small intestine may still have negative results, even with the accurate location of the malformed vessels because of the quick excretion of the contrast agent.

**Case summary:**

A 69-year-old woman presented with recurrent melena lasting for over 3 years, including a recent aggravation 2 days prior to admission. DSA revealed abnormal contrast uptake in the distal part of the first branch of the left superior mesenteric artery. Enhanced computed tomography (CT) scan confirmed the presence of vascular malformations in the small intestine. DSA and methylene blue staining were further utilized in a hybrid operating room to locate the vascular malformation of the small intestine. Laparoscopy-assisted synchronous resection of the stained intestine was performed simultaneously, effectively resolving the intestinal bleeding associated with the malformed vessels. The patient was discharged on postoperative day 5, without complication. She experienced no complications, such as intestinal fistula and hematochezia, at the 6-month follow-up.

**Conclusion:**

With increased experience, laparoscopy and methylene blue staining angiography may offer a safe and feasible method for synchronous resection of small intestine vascular malformations.

## Introduction

The incidence of small intestinal bleeding is low, and it is even rarer for small intestinal vascular malformations to serve as the underlying cause (1). In clinical practice, an unclear diagnosis leads to prolonged illness and poor prognosis. The location of bleeding in the small intestine makes laparoscopic surgery difficult, which is commonly carried out via the open method (2, 3). Even when malformed vessels can be located using angiography, the diseased small intestine cannot be found during a heterochronous operation, resulting in an unsuccessful operation (4). The present report describes and highlights the safety and feasibility of a simultaneous method of laparoscopy combined with methylene blue staining angiography for accurate synchronous resection of the small intestine with vascular malformations performed in the hybrid operating room. This type of synchronous resection is relatively rare, and only a few studies on double-balloon enteroscopy-guided operation have been published (5). The present report introduces the technical aspects of the method in detail.

## Case presentation

**Figure 1:**
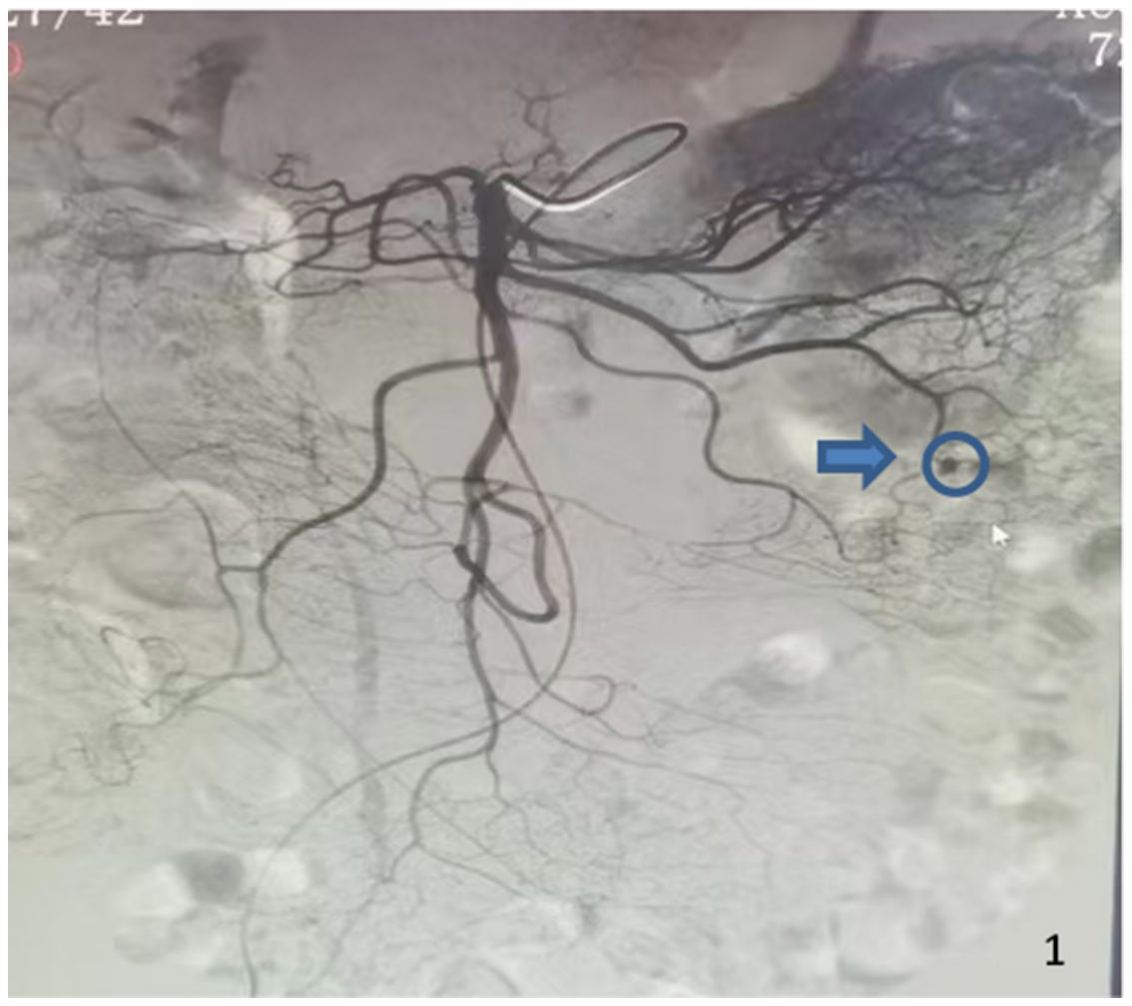
Preoperative DSA imaging shows abnormal contrast uptake at the distal part of the first branch of the left superior mesenteric artery (blue arrow and circle).

A 69-year-old woman with a history of hepatitis B virus-related cirrhosis, maintained with long-term entecavir therapy, was admitted for recurrent hematochezia lasting over 3 years, including a recent aggravation 2 days prior to admission. A physical examination revealed a flat, non-tender abdomen without liver and spleen and an absence of shifting dullness. The rest of the abdominal examination was unremarkable. A routine blood test showed a hemoglobin level of 67 g/L. A computed tomography (CT) scan confirmed hepatic cirrhosis, splenomegaly, abdominal varices, left perirenal varices with focal tumor-like dilation, multiple retroperitoneal lymphadenopathies, and gallstones with cholecystitis. Digital subtraction angiography (DSA) revealed abnormal contrast uptake at the distal part of the first branch of the left superior mesenteric artery, with early imaging of mesenteric veins (Figure 1). Multi-angle angiography and XPER-CT scan confirmed the presence of vascular malformations in the small intestine. Capsule endoscopy performed during the hospital stay did not reveal significant pathological findings. The main diagnoses included the following: (1) gastrointestinal hemorrhage secondary to small intestinal vascular malformation, (2) hepatitis B virus-related cirrhosis, and (3) splenomegaly.

**Figure 2:**
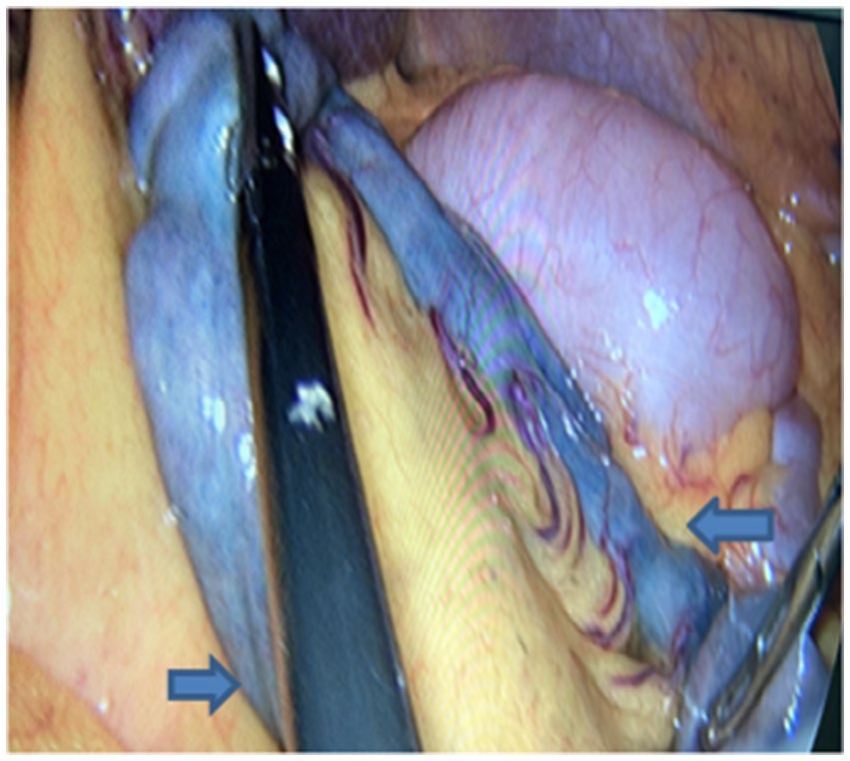
Postoperative exploration reveals ~10 cm of jejunum stained with methylene blue.

**Figure 3:**
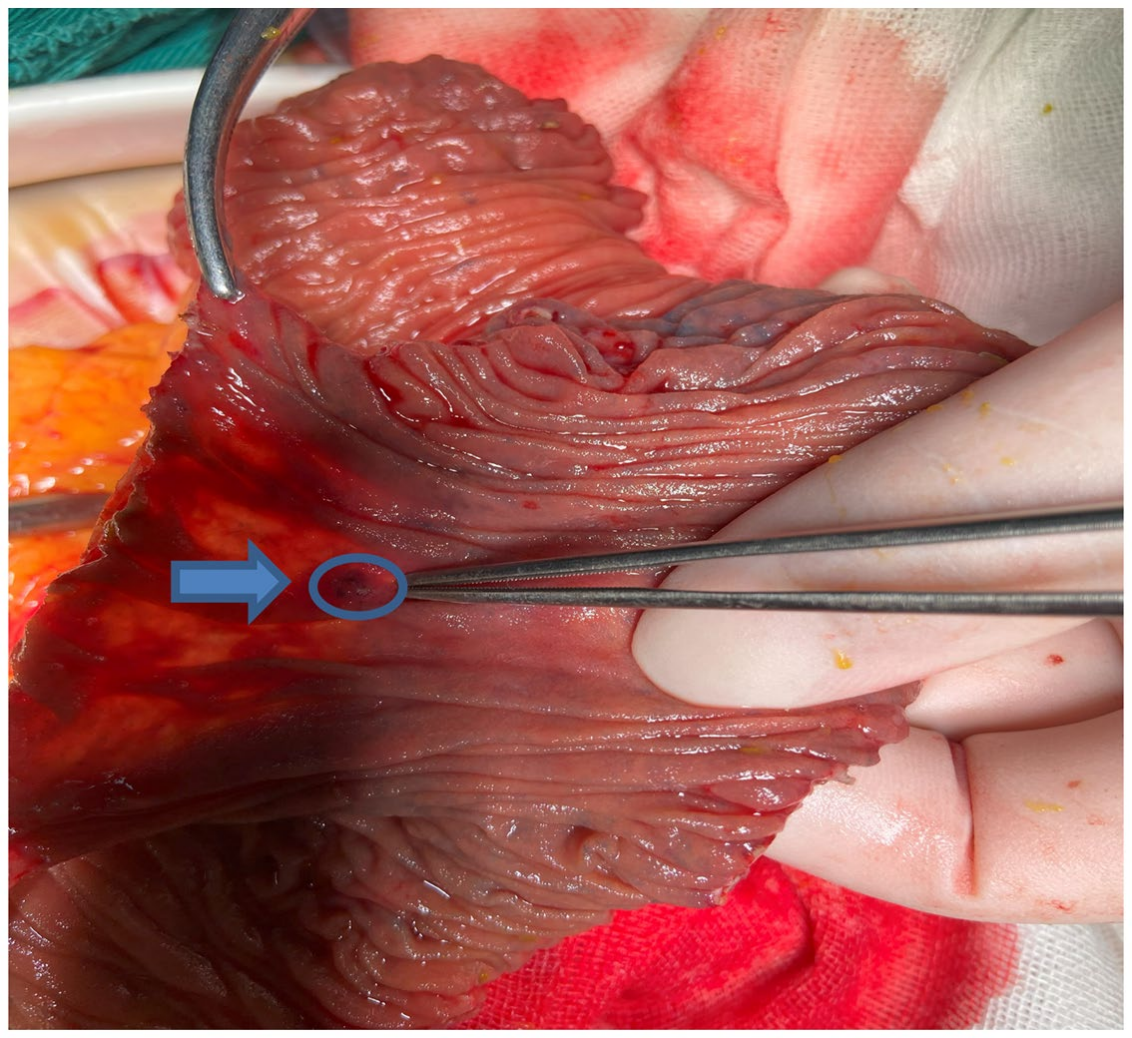
1-mm vascular lesion found in the removed jejunal mucosa (blue arrow and circle).

**Figure 4:**
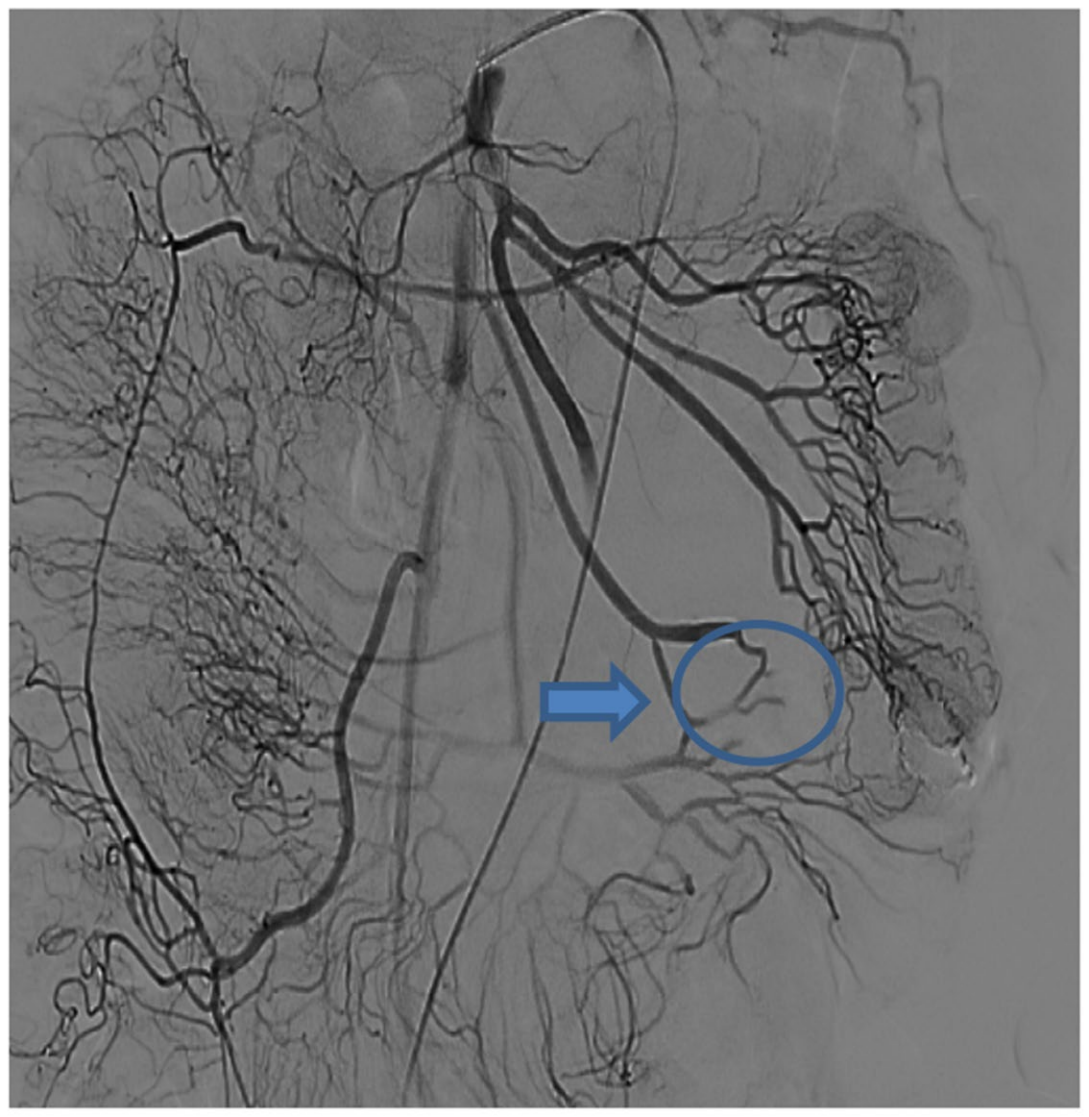
Postoperative DSA imaging shows the successful ligation of the vascular malformation, with no additional anomalies(blue arrow and circle).

DSA performed in the hybrid operating room initially identified a distal malformed vessel located in the first branch of the left superior mesenteric artery. An angiographic tube was placed near the vascular malformation, followed by standard skin preparation and draping procedures. A 10-mm trocar was inserted below the umbilicus, accompanied by the placement of three 5-mm trocars in the right and left parts of the abdomen. Intestinal forceps were used to straighten the jejunum, and approximately 3 mL of methylene blue (2 mL/piece) was injected through the tube, rapidly staining parts of the jejunum (Figure 2). The injection rate of methylene blue was 1 mL every 10 s. The stained jejunum was clamped at both ends, and a 6-cm incision was made along the left rectus abdominis. Approximately 10 cm of the stained portion was pulled out, revealing a 1-mm vascular lesion in the mucosa (Figure 3). Postoperative DSA verified the successful ligation of the vascular malformation, with no additional anomalies (Figure 4). Postoperative recovery was successful, and the patient was discharged on postoperative day 5. No complications, such as intestinal fistula and hematochezia, were noted at the 6-month follow-up. The blood tests indicated that the hemoglobin levels had returned to normal. Although the patient still needs to visit for follow-up for a longer time period, relapse is less likely to occur.

### Pathological findings

Submucosal vascular hyperplasia in the jejunum was observed, characterized by dilation, distortion, congestion, and bleeding, which was consistent with a diagnosis of vascular malformation. One lymph node in the mesentery showed chronic inflammation.

## Discussion

Vascular malformation is an uncommon disease in adults that affects the small intestine (2, 6, 7). Clinically, it typically manifests itself as recurrent hematochezia. Bleeding from the small intestine is often insidious and tends to be prolonged (3, 6, 8, 9). Vascular malformations are responsible for approximately 30–40% of cases of small intestinal bleeding (1, 9). No evidence of cirrhosis-related portal hypertensive gastropathy or enteropathy was identified in this patient by routine gastrointestinal endoscopy prior to surgery. The female patient described in the present case had hepatitis B virus-related cirrhosis and portal hypertension that may have promoted the occurrence of small intestinal vascular malformation bleeding. Currently, there are few studies on the synchronous resection of small intestinal vascular malformations using laparoscopy and methylene blue staining angiography worldwide. Therefore, the safety and feasibility of this combined operation were further emphasized in the present report.

A CT scan is highly valuable for detecting acute massive hemorrhage in the small intestine. However, the detection of vascular malformations associated with chronic blood loss remains a challenge. Capsule endoscopy is a minimally invasive technique for gastrointestinal evaluation. It has a high diagnostic rate (60–80%) for small intestinal lesions (10–13), particularly for small intestinal tumors, inflammation, and ulcerations. However, this method exhibits low sensitivity in detecting non-bleeding arteriovenous malformations in the small intestine (14, 15). In this case, we also had no positive findings. Double balloon endoscopy (DBE) overcomes the drawback of capsule endoscopy, especially its inability to perform tissue biopsy and therapy (5, 16). Recent studies have reported that a methylene blue injection has been used during preoperative DBE to precisely locate submucosal lesions (17). However, the combination of methylene blue staining and DBE requires highly skilled operators, involves a prolonged surgical duration, and entails high costs for both equipment and operation, thereby posing challenges for widespread clinical application (18).

By integrating multi-modal imaging and minimally invasive surgery, the hybrid operating room enables real-time image-guided intervention during surgery. Its core equipment includes a DSA system, a laparoscopic system, and other minimally invasive tools. This setup reduces intraoperative decision delays and enhances precision in complex operations, facilitating synchronous surgery. DSA is widely regarded as the gold standard for small intestinal vascular malformations due to its superior capability of precisely locating the lesion and identifying the contrast agent. After a definitive diagnosis of intestinal vascular malformation by DSA, it was initially decided not to embolize due to potential intestinal avascular necrosis. Boullier et al. (19) also reported that DSA can embolize the malformed vessels to stop bleeding, although potential complications included intestinal ischemia and necrosis.

To date, no consensus has been reached regarding the choice of contrast agents. Hyo et al. presented compelling evidence regarding the efficacy of indocyanine green (ICG) in enhancing the visualization of small intestinal vascular malformations (20). Qiao H et al. reported on the use of methylene blue dye injection for the identification of recurrent small intestinal bleeding (21). In the present study, methylene blue was also successfully used for visualization. Methylene blue is cheaper than ICG, and its staining range is limited, reducing the risk of widespread dye diffusion and preventing incorrect lesion localization. The adverse effects of methylene blue include gastrointestinal symptoms (5–20%), local irritations (10–30%) and allergic reactions (<1%) (22). However, this female patient did not experience any of these effects.

At present, surgical resection represents an effective treatment for vascular malformations in the small intestine (23). However, this approach needs to overcome the technical challenges associated with accurate lesion location. Traditional methods have used spring pins or guide wires as locating devices and have been combined with either tactile feedback or C-arm fluoroscopic imaging for lesion identification (24, 25). However, many difficulties have been noted in clinical practice, particularly in cases where a guide wire is not detectable due to mesenteric hypertrophy or when it is displaced during operation. The present study used methylene blue staining in combination with DSA in a hybrid operating room to ensure the precise location of vascular malformations in the small intestine. A laparoscopic resection of the identified segmental intestine was performed simultaneously. After removal, DSA was repeated to confirm the complete excision of vascular malformations. Furthermore, an incision was made in the stained intestine to verify the bleeding vascular foci within the mucosa.

The following aspects need to be considered during the operation:

First, the angiography tube must be positioned as close as possible to the intestinal vascular malformation to minimize the range of methylene blue staining. This step enables both the rapid and precise location of the vascular malformation while avoiding excessive resection of unaffected tissue.

Second, the preoperative assessment of the vascular malformation is essential to ensure its precise location and to determine whether the lesion is situated in the jejunum or ileum and in the upper or lower part of the jejunum. The present evaluation was based on the distribution patterns of jejunal and ileal arterial branches and their corresponding vascular arches. The anticipated segmental intestine was straightened using intestinal forceps during the procedure. Immediate clamping of both ends of the stained intestine was important after methylene blue staining.

Finally, DSA imaging should be repeated after the resection of the stained segment to confirm the complete removal of the vascular malformation. The corresponding arterial branch associated with the vascular malformation was found to be severe.

In conclusion, the application of methylene blue staining in DSA imaging facilitates the determination of a precise vascular malformation location in the small intestine. Simultaneous laparoscopy-assisted resection in a hybrid operating room demonstrates both the safety and feasibility of the method with definitive clinical advantages, including a one-time operation to cure chronic small intestinal bleeding and treatment to improve surgical accuracy. The only drawback of this method is radiation due to multiple scans. This synchronous operation is worthy of further clinical application and promotion.

## Authors note

Core tip: The present report describes a patient who underwent precise synchronous resection of vascular malformations originating from the small intestine performed in the hybrid operating room, including both laparoscopy and methylene blue staining angiography. There are a few similar reports worldwide (26).

## Data Availability

The datasets presented in this study can be found in online repositories. The names of the repository/repositories and accession number(s) can be found in the article/supplementary material.

## References

[1] Othman B Tan JYC Friedman A Steel M. Resistive small bowel bleeding secondary to an arteriovenous malformation. ANZ J Surg. (2023) 93:376–8. doi: 10.1111/ans.17815, PMID: 35621035

[2] Saeed S Naz S Iqbal A Irfan M Khan S Jaiswal V. Arteriovenous malformations in proximal part of ileum: a case report. JNMA J Nepal Med Assoc. (2021) 59:706–8. doi: 10.31729/jnma.6929, PMID: 34508492 PMC9107869

[3] Dunphy L Ford J. Arteriovenous malformation of the small intestine presenting with a transfusion-dependent anaemia in pregnancy. BMJ Case Rep. (2023) 16:e251653. doi: 10.1136/bcr-2022-251653, PMID: 36889804 PMC10008215

[4] Dabora AA Nogoud A Abdulsakhi M Rafei A Khalifa HA. Jejunal angiodysplasia: surgery can be life-saving - a case report. Ann Med Surg (Lond). (2024) 86:2204–7. doi: 10.1097/MS9.0000000000001799, PMID: 38576924 PMC10990319

[5] Nomura K Shibuya T Terai Y Omu R Arii S Yuzawa A. Small intestinal arteriovenous malformation treated by double-balloon endoscopy. Intern Med. (2024) 63:2131–5. doi: 10.2169/internalmedicine.2588-23, PMID: 38104993 PMC11358740

[6] Pohl J Kahya S Heise M Faiss S Müller N. Episodic bleeding events due to arteriovenous malformation (AVM) in the region of the distal ileum in a young male patient. Innere Medizin. (2024) 65:172–5. doi: 10.1007/s00108-023-01556-4, PMID: 37542011

[7] Chen Z Liu Z Tang J. An Unruptured Jejunal aneurysm in a female patient with melena caused by arteriovenous malformation. J Gastrointest Surg. (2021) 25:1073–5. doi: 10.1007/s11605-020-04777-2, PMID: 32869143

[8] Fujimori S. Tranexamic acid may be a useful pharmacotherapy for endoscopically resistant small bowel angiodysplasia. World J Gastroenterol. (2023) 29:1131–8. doi: 10.3748/wjg.v29.i7.1131, PMID: 36926669 PMC10011953

[9] Ávila VB Espinel Diez J Jiménez Palacios M. Jejunal Dieulafoy's lesion as a cause of difficult-to-manage obscure gastrointestinal bleeding. Combined endoscopic treatment. Rev Esp Enferm Dig. (2024) 116:709. doi: 10.17235/reed.2024.10176/2023, PMID: 38235668

[10] Nnocenti T Dragoni G Roselli J Macrì G Mello T Milani S. Non-small-bowel lesions identification by capsule endoscopy: a single Centre retrospective study. Clin Res Hepatol Gastroenterol. (2021) 45:101409. doi: 10.1016/j.clinre.2020.03.011, PMID: 32245690

[11] Yang W Li Z Liu R Tong X Wang W Xu D. Application of capsule endoscopy in patients with chronic and recurrent abdominal pain: abbreviated running title: capsule endoscopy in abdominal pain. Med Eng Phys. (2022) 110:103901. doi: 10.1016/j.medengphy.2022.103901, PMID: 36241495

[12] Chu Y Huang F Gao M Zou DW Zhong J Wu W. Convolutional neural network-based segmentation network applied to image recognition of angiodysplasias lesion under capsule endoscopy. World J Gastroenterol. (2023) 29:879–89. doi: 10.3748/wjg.v29.i5.879, PMID: 36816625 PMC9932427

[13] Ghoshal UC Mishra P Mathur A Reddy SP Fatima B Misra A. Capsule endoscopy for obscure gastrointestinal bleed in the tropics: a single-center experience on 350 patients. Indian J Gastroenterol. (2024) 43:1045–55. doi: 10.1007/s12664-024-01526-0, PMID: 38517665

[14] Phillips F Beg S. Video capsule endoscopy: pushing the boundaries with software technology. Transl Gastroenterol Hepatol. (2021) 6:17. doi: 10.21037/tgh.2020.02.01, PMID: 33409411 PMC7724185

[15] Pennazio M Rondonotti E Despott EJ Dray X Keuchel M Moreels T. Small-bowel capsule endoscopy and device-assisted enteroscopy for diagnosis and treatment of small-bowel disorders: European Society of Gastrointestinal Endoscopy (ESGE) guideline - update 2022. Endoscopy. (2023) 55:58–95. doi: 10.1055/a-1973-3796, PMID: 36423618

[16] Ye X Liao Y Deng T. Comparison of the positive rate and diagnostic value of capsule endoscopy and double-balloon Enteroscopy in small bowel disease: a retrospective cohort analysis. Arch Iran Med. (2021) 24:218–23. doi: 10.34172/aim.2021.33, PMID: 33878880

[17] Tohma T Okabe Y Ushio M Saito M. Arteriovenous malformation of small intestine successfully treated by double-balloon enteroscopy and laparoscope-assisted surgery. J Surg Case Rep. (2022) 12:1–3. doi: 10.1093/jscr/rjac606, PMID: 36601092 PMC9803965

[18] Shinozaki S Yamamoto H Yano T. Comparison of double-balloon endoscopy and capsule endoscopy for obscure gastrointestinal bleeding: a systematic review and meta-analysis. Dig Endosc. (2020) 32:823–33.31957073

[19] Boullier M Fohlen A Viennot S Alves A. Gastrointestinal bleeding of undetermined origin: what diagnostic strategy to propose? J Visc Surg. (2023) 160:277–85. doi: 10.1016/j.jviscsurg.2023.05.006, PMID: 37344277

[20] Hyo T Matsuda K Tamura K Iwamoto H Mitani Y Mizumoto Y. Small intestinal arteriovenous malformation treated by laparoscopic surgery using intravenous injection of ICG: case report with literature review. Int J Surg Case Rep. (2020) 74:201–4. doi: 10.1016/j.ijscr.2020.08.038, PMID: 32890897 PMC7481494

[21] Qiao H Ding N Chen X. Methylene blue localization is a procedure for the laparoscopic surgery: a worthy recurrent small intestinal bleeding. Asian J Surg. (2023) 46:3326–7. doi: 10.1016/j.asjsur.2023.03.052, PMID: 36964065

[22] Kang S Lee J Park H. Anaphylactic shock following methylene blue injection for sentinel lymph node mapping: a case report. J Clin Anesth. (2025) 78:110345

[23] Shiraishi T Kunizaki M Takaki H Horikami K Yonemitsu N Ikari H. A case of arteriovenous malformation in the inferior mesenteric artery region resected surgically under intraoperative indocyanine green fluorescence imaging. Int J Surg Case Rep. (2022) 92:106831. doi: 10.1016/j.ijscr.2022.106831, PMID: 35176584 PMC8857500

[24] Arieira C Magalhães R Dias de Castro F Boal Carvalho P Rosa B Moreira MJ. Small bowel Angioectasias Rebleeding and the identification of higher risk patients. Dig Dis Sci. (2021) 66:175–80. doi: 10.1007/s10620-020-06137-1, PMID: 32072436

[25] Sandhu S Gross J Barkin JA. Small bowel bleeding due to vascular lesions: pathogenesis and management. Curr Gastroenterol Rep. (2025) 27:37. doi: 10.1007/s11894-025-00989-1, PMID: 40481967 PMC12145302

[26] Hamada Y Umeda Y Ikenoyama Y Shigefuku A Yukimoto H Nakamura M. Obscure gastrointestinal bleeding caused by a small intestinal lymphatic-venous malformation: a case report with a literature review. Intern Med. (2023) 62:387–91. doi: 10.2169/internalmedicine.9733-22, PMID: 35732456 PMC9970798

